# Laparoscopic repair of a Spigelian hernia with appendicitis *in situ*

**DOI:** 10.1111/ans.17930

**Published:** 2022-07-22

**Authors:** Guy H. M. Stanley, Tristan Gilliland, Matthew W. Trinder, Enoch Wong, Yu Xin Liew, Jennifer Ryan

**Affiliations:** ^1^ Department of surgery Fiona Stanley Hospital Murdoch Western Australia Australia; ^2^ Department of Surgery, Medical school University of Western Australia Crawley Western Australia Australia

A 71‐year‐old male presented with a 15‐hour history of migratory right lower quadrant (RLQ) abdominal pain. He had no nausea, vomiting, diarrhoea, dysuria or recent illnesses. His medical history was significant for a laparoscopic‐assisted radical prostatectomy (2016) and laparoscopic right inguinal & femoral hernia repair (2011). A colonoscopy 2 years prior was unremarkable.

Examination revealed a soft abdomen, no masses but tenderness to light palpation in the RLQ. Vital signs were normal. Full blood count, urea, electrolytes & creatinine, liver function tests and lipase were normal, while C‐reactive protein was elevated at 11 mg/L.

Computed tomography (CT) of the abdomen and pelvis with portal venous contrast reported a right‐sided, ventrolateral or Spigelian hernia containing inflamed appendix with tip located between external oblique aponeurosis and internal oblique muscles (Fig. 1). We performed a confirmatory ultrasound, which suggested uncomplicated appendicitis in a spigelian hernia (Fig. 2).

**Fig. 1:**
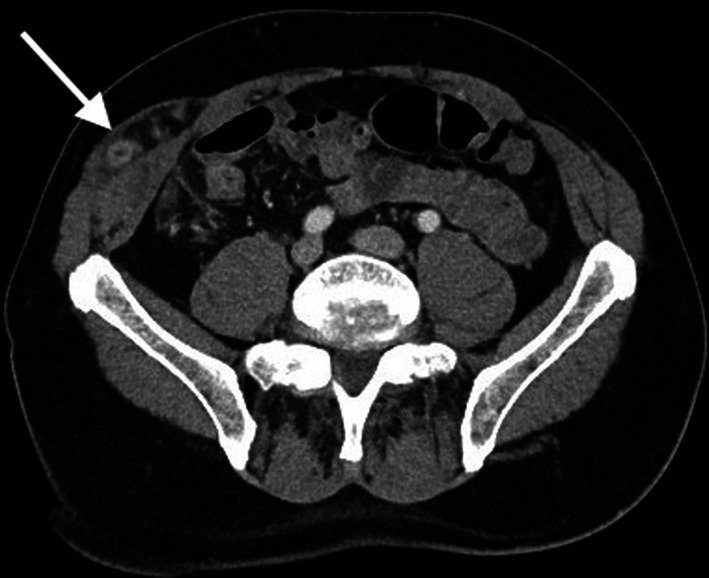
Axial computed tomography of the abdomen and pelvis showing appendicitis within a Spigelian hernia.

**Fig. 2:**
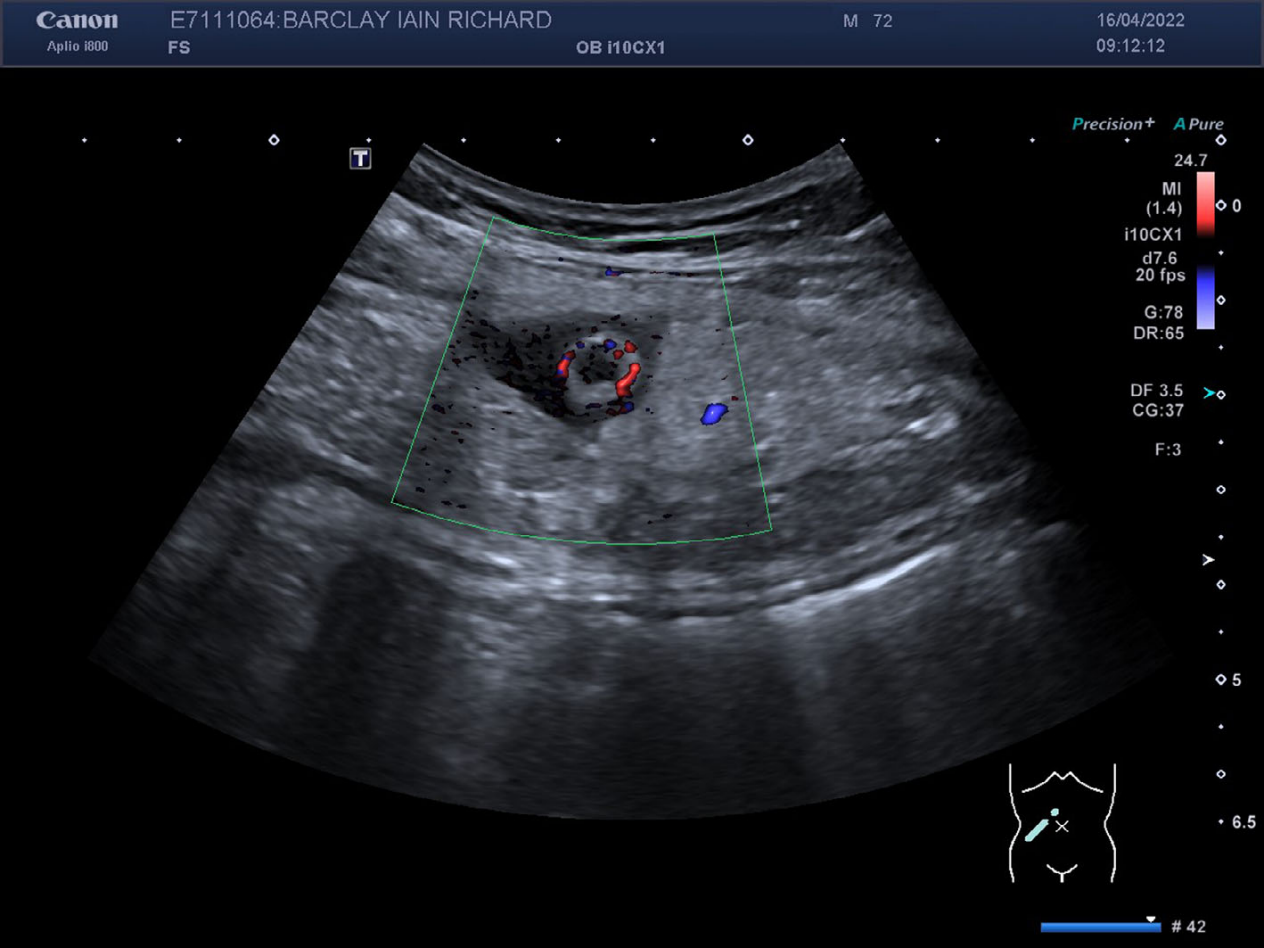
An abdominal ultrasound confirming appendicitis within a spigelian hernia.

The patient underwent laparoscopic appendicectomy and hernia repair. Intra‐operative findings showed a hernial gate 5 mm wide, consistent with a Spigelian hernia and incarcerated appendicitis within (Fig. 3). The appendix was removed via an Endo Catch™ with Endo Close™ repair of the hernia using interrupted 0 PROLENE sutures (MEDTRONIC, Minneapolis, US). The patient was discharged the next day without complications. Histology confirmed acute, suppurative appendicitis. Informed consent was obtained from the patient for publication of this case.

**Fig. 3:**
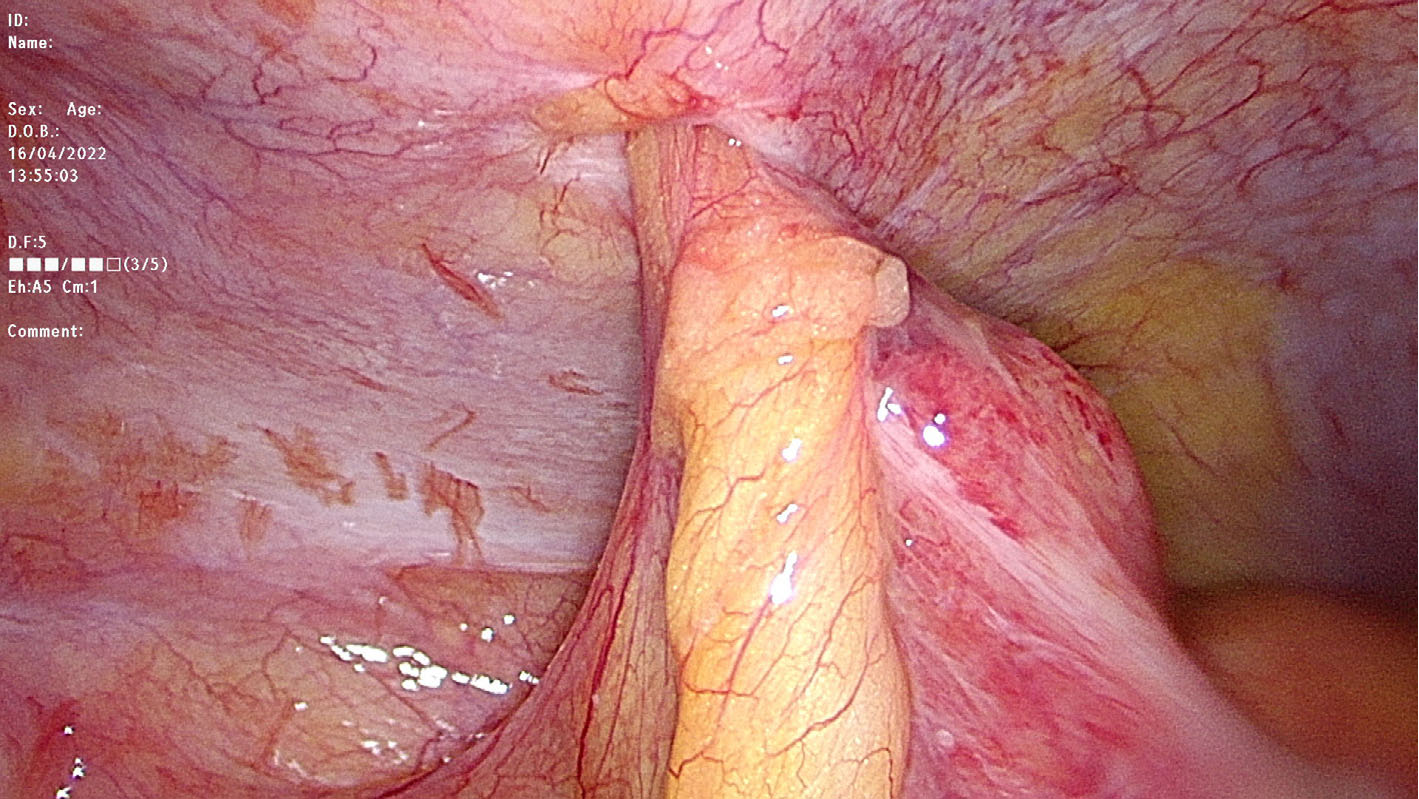
A laparoscopic view of the appendix, incarcerated within a spigelian hernia.

Appendicitis is common, with a 7%–8% lifetime risk,^1^ while Spigelian hernias are relatively rare, accounting for <2% of all hernias.^2^ The rate of hernial appendicitis is 0.008%, most common in inguinal and femoral hernias.^3^ 18 published studies reported appendices within Spigelian hernias.

Light (2013) reported Spigelian hernia diagnosis with ultrasound had sensitivity of 90% and positive predictive value of 100%, while CT was 100% & 100%, respectively.^4^ 12/18 (67%) studies obtained preoperative diagnosis from CT; the rest were diagnosed intraoperatively. In our case, a CT abdomen (2016) showed no hernia pre‐prostatectomy, while contemporary CT, ultrasound, and intraoperative findings suggested an acquired Spigelian. Confirmatory ultrasound may have been redundant.

Spieghel identified the semilunar line (*linea Spigeli*) in 1645. Klinkosch (1764) named the eponymous hernia resulting from its defect. These can be acquired or congenital (hypothetically from abdominal muscle aponeurotic weakness during mesenchyme development in the somatopleura^2^). In our case, an incisional hernia through a laparoscopic port site was a significant differential however, the hernia was separate from previous incisions.

Spigelian hernias are predisposed to incarceration due to a typically small (<2 cm) hernial gate with a sharp fascial edge.^5^, ^6^, ^7^ The rate of strangulation was reported as 2%–14%.^8^ In our case, a narrow hernial gate caused extraluminal compression leading to impaired venous return and ischaemia.

The literature reports open surgery in 11/18 (61%) cases and laparoscopic intra‐abdominal approaches in 5/18 (28%). In 4/18 (28%), surgeons used mesh to repair the hernia at the primary operation. Only 3/18 (17%) reported studies described finding a normal appendix.

This case demonstrates the anatomy, workup and management of appendicitis within a Spigelian hernia and underlines the value of preoperative imaging and laparoscopic repair.

The corresponding author is the recipient of the Cynthia Banham Burn Injury Research Fellowship [Ian Potter Foundation; 2021] and received the Australian Government Research Training Program Fees Offset Scholarship for a higher degree [University of Western Australia; 2022].

This case has not been reported, presented or published before.

## Author contributions

**Guy H. M. Stanley:** Data curation; formal analysis; investigation; project administration; writing – original draft; writing – review and editing. **Tristan Gilliland:** Data curation; formal analysis; investigation; project administration; writing – original draft; writing – review and editing. **Matthew W. Trinder:** Conceptualization; methodology; supervision; validation; writing – review and editing. **Yu Xin Liew:** Writing – review and editing. **Enoch Wong:** Methodology; supervision; validation; writing – review and editing. **Jennifer Ryan:** Supervision; validation; writing – review and editing.
